# Multiomics Integration Prioritizes *ZFP36L1* as a Candidate Susceptibility Gene Associated With Inflammatory and Angiogenic Pathways in Diabetic Retinopathy

**DOI:** 10.1155/jdr/4312504

**Published:** 2026-06-19

**Authors:** Ruiqing Dong, Xiaoli Hui, Chenwen Luo, Shengnan Chen, Ning Gao, Mingqian He, Bingyin Shi, Wei Qiang

**Affiliations:** ^1^ Department of Endocrinology and Metabolism, The First Affiliated Hospital of Xi′an Jiaotong University, Xi′an, China, xjtu.edu.cn; ^2^ Department of Endocrinology and Second Department of Geriatrics, The First Affiliated Hospital of Xi′an Jiaotong University, Xi′an, China, xjtu.edu.cn; ^3^ Department of Clinical Medicine, Xi′an Medical University, Xi′an, China, xjtu.edu.cn; ^4^ Department of Critical Care Nephrology and Blood Purification, The First Affiliated Hospital of Xi′an Jiaotong University, Xi′an, China, xjtu.edu.cn; ^5^ Department of Ophthalmology, The First Affiliated Hospital of Xi′an Jiaotong University, Xi′an, China, xjtu.edu.cn

**Keywords:** diabetic retinopathy, inflammation, Mendelian randomization, NF-*κ*B signaling, single-cell RNA sequencing, *ZFP36L1*

## Abstract

**Background:**

Diabetic retinopathy (DR) is a prevalent microvascular complication of diabetes and a leading cause of blindness despite available therapies, underscoring the need for novel therapeutic targets.

**Methods:**

Integrated multiomics analysis combining (1) exploratory single‐cell RNA sequencing of rat retinal tissues (three DRs vs. two controls), (2) Mendelian randomization (MR) using whole‐blood eQTL data and DR GWAS data (14,584 cases vs. 202,082 controls), and (3) supportive RT‐qPCR validation in peripheral‐blood clinical samples (30 DRs vs. 30 controls).

**Results:**

(1) Genetic causality was as follows: MR analysis demonstrated *ZFP36L1* as a novel DR risk gene (OR = 1.156, 95% CI = 1.054–1.269, *p* = 0.002), with significant upregulation in patient blood samples (*p* < 0.0001). (2) Inflammatory regulation was as follows: *ZFP36L1* showed strong correlations with proinflammatory markers (TNF‐*α* and IL‐6) and immune cell infiltration (parainflammation and Tregs), while negatively correlating with B cells. (3) Pathway mechanisms were as follows: Functional enrichment analyses suggested potential links between *ZFP36L1* and NF‐*κ*B–related inflammatory signaling as well as TGF‐*β*–related angiogenic pathways. Despite its known VEGF mRNA destabilizing function, we observed a positive *ZFP36L1*–*VEGF* correlation (*r* = 0.180, *p* < 0.001), an unexpected finding under diabetic conditions.

**Conclusions:**

*ZFP36L1* is a candidate DR susceptibility gene whose genetically regulated expression in blood is associated with DR risk. Retinal single‐cell and pathway analyses provide supportive, hypothesis‐generating evidence that *ZFP36L1* may be linked to inflammatory and angiogenic processes relevant to DR. Further retina‐specific and functional studies are required to determine whether *ZFP36L1* directly regulates retinal inflammation or angiogenesis in human DR.

## 1. Introduction

Diabetic retinopathy (DR) remains one of the most prevalent microvascular complications of diabetes, affecting approximately 40% of patients within a decade of diagnosis and contributing to 4.2 million cases of blindness worldwide [1–3]. Current therapies, including anti‐VEGF agents and laser photocoagulation, demonstrate limited efficacy (60%–70% response rates) and fail to address the underlying inflammatory and neurodegenerative components of DR [4–6]. This therapeutic gap highlights the critical need for unbiased identification of novel molecular targets that drive DR progression, particularly those that bridge genetic susceptibility with functional pathways.

The pathological complexity of DR—which includes pericyte loss, breakdown of the blood–retinal barrier, and chronic retinal inflammation—has historically hindered target discovery [7, 8]. Recent advances in single‐cell RNA sequencing (scRNA‐seq) now enable cell type–specific resolution of transcriptional changes in diabetic retinas. Meanwhile, Mendelian randomization (MR) analysis of large‐scale genome‐wide association study (GWAS) data provides causal inference linking gene expression to disease risk [9–12]. Crucially, integrating these approaches provides a hypothesis‐neutral framework for prioritizing functionally relevant genes, as demonstrated by recent successes in renal disease research [13]. Recent studies have begun applying multiomics to DR, identifying new candidate genes and pathways [14–16]. This paradigm shift—from candidate gene to data‐driven strategies—is particularly valuable for DR, a condition whose mechanistic heterogeneity demands systematic discovery approaches.

We combined scRNA‐seq of rat retinal samples from a diabetic rat model (GSE209872) with MR analysis of GWAS data to systematically identify genes associated with DR. This computational prioritization approach identified *ZFP36L1* as a candidate gene that showed both differential expression in DR retinas and genetic evidence supporting a causal association with DR risk (OR = 1.156, *p* = 0.002). The bioinformatic predictions were further supported by RT‐qPCR validation, which demonstrated elevated *ZFP36L1* expression in peripheral blood samples from DR patients compared with healthy controls (*p* < 0.0001), as well as by correlation analysis with established DR‐related markers.

## 2. Materials and Methods

### 2.1. Data Collection

We retrieved the publicly available scRNA‐seq dataset GSE209872 from the Gene Expression Omnibus (GEO) database (https://www.ncbi.nlm.nih.gov/geo/info/datasets.html) and selected three DR samples together with two intact control samples for scRNA‐seq. We used GSE209872, which contains five rat retinal specimens (controls: 0 week, *n* = 2; diabetic/DR model: 2/4/8 weeks, *n* = 3). Because these data are from a rat model, human clinical staging (nonproliferative diabetic retinopathy [NPDR]/proliferative diabetic retinopathy [PDR]) is not applicable; samples are defined by time after diabetes induction. We obtained blood eQTL summary statistics from eQTLGen and collected DR GWAS data (finn–b–DM_RETINOPATHY) from the GWAS Catalog (14,584 cases; 202,082 controls). In the GWAS layer, the FinnGen endpoint represents a clinically coded DR phenotype that may aggregate heterogeneous manifestations of DR. In the peripheral‐blood validation cohort, DR was defined clinically, but the sample size did not permit robust stage‐stratified analysis. Accordingly, the three analytical layers should be interpreted as complementary but not identical representations of DR. For external transcriptomic validation, we downloaded GSE94019, GSE60436, and GSE102485, which are based on human PDR‐related tissues.

### 2.2. ScRNA‐Seq Data Analysis and Enrichment Analysis

All scRNA‐seq analyses were executed by leveraging the standard workflow provided by the Seurat toolkit in R. Cells failing to satisfy nFeature RNA between 350 and 3000 or exhibiting mitochondrial gene proportions ≥ 20*%* were discarded. After scaling raw counts with the LogNormalize approach, we performed principal component analysis for dimension reduction, followed by uniform manifold approximation and projection (UMAP) for low‐dimensional representation. No SCTransform normalization or Harmony batch correction was performed, as all five retinal samples originated from the same dataset and were processed together. Clusters were then assigned biological identities according to canonical marker genes. Differentially expressed genes were defined by an absolute log‐fold change exceeding 1 and an adjusted *p* value cutoff of 0.05 when comparing case versus control cells. Functional interpretation of candidate genes was carried out with the R package ClusterProfiler, facilitating a systematic survey of their biological associations. Gene Ontology (GO) enriched pathways with both *p* value and *q* value less than 0.05 were considered significant categories.

### 2.3. MR

Gene expression (eQTL data at summary level) was considered as exposure, whereas DR (GWAS data at summary level) was used as the outcome variable. The outcome screened through the MR‐Base database (*N* = 216,666) was extracted in the GWAS summary data for relevant causality in eQTL, and the genetic instrumental variables used for MR analysis obeyed three MR hypotheses: (1) SNPs are strongly associated with exposure at a genome‐wide significance level (*p* < 1 × 10^−8^), (2) SNPs are not associated with any potential confounders and were independent of each other to avoid bias caused by linkage disequilibrium (*r* ^2^ < 0.001, clumping distance = 10,000 kb), and (3) SNPs affect outcome only through exposure. The reliability of causality was assessed using the Wald ratio method for single IV MR and inverse‐variance weighted (IVW), MR‐Egger, weighted median method, and weighted mode for two or more SNPs for all cis‐ and some cross‐region gene expression in whole blood on DR. Finally, the screened causal relationships were validated and analyzed by heterogeneity (Cochran′s *Q* test under IVW), pleiotropy analysis, and leave‐one‐out sensitivity analysis. Heterogeneity among SNP‐specific causal estimates was assessed with Cochran′s *Q*; a *p* value ≤ 0.05 suggested heterogeneity, prompting adoption of the random‐effects IVW MR model. Transcriptome data (GSE94019, GSE60436, and GSE102485) were used to validate expression differences in the selected genes.

### 2.4. Immune Cell Infiltration Analysis

Transcriptome‐based immune enrichment analysis was performed to estimate the relative abundance of 29 immune cell subsets from bulk expression data. This approach was selected because it enables comparative assessment of immune‐related transcriptional signals when direct immunophenotyping data are unavailable. Associations between gene expression and inferred immune‐cell scores were evaluated using Spearman correlation.

### 2.5. Gene Set Enrichment Analysis (GSEA) and Gene Set Variation Analysis (GSVA)

GSEA was performed to compare pathway differences between the high‐ and low‐expression groups. The background gene sets were obtained from the Molecular Signatures Database (MSigDB) (Version 7.0). Significantly enriched gene sets were ranked according to enrichment score, and pathways with adjusted *p* < 0.05 were considered significant. GSVA was used as a nonparametric, unsupervised method to transform gene‐level expression profiles into pathway‐level scores, thereby assessing potential biological‐function differences across samples.

### 2.6. Regulation Analysis of Key Gene and Related Analysis in Single‐Cell Data

We explored the regulatory relationships between transcription factors and key gene via Cistrome DB, in which the genome file was set to hg38 and the transcription start site was set to 10 kb, and rendered the resulting networks in Cytoscape. DR‐linked genes were subsequently retrieved from the GeneCards portal (https://www.genecards.org/), and their expression patterns were correlated with those of the core gene set. Finally, we analyzed the expression of key gene, coexpression networks, and correlation of immune/metabolic pathways in single‐cell data.

### 2.7. Collection of Clinical Samples and RT‐qPCR

Peripheral blood samples were collected from two groups: healthy controls (*n* = 30) recruited from the Health Examination Center of the First Affiliated Hospital of Xi′an Jiaotong University and DR patients (*n* = 30) from the Department of Endocrinology and Metabolism at our hospital. Ethical approval was obtained from the Ethics Committee of the First Affiliated Hospital of Xi′an Jiaotong University, with informed consent obtained from all participants (Approval No. XJTU1AF2023LSK–458).

Peripheral blood samples were homogenized in 500 *μ*L of RNAiso Plus. The mixture was incubated at room temperature for 5 min and then centrifuged at 12,000 × g for 5 min at 4°C. The supernatant was transferred to a new 1.5‐mL tube, and 100 *μ*L of chloroform was added. The tube was shaken vigorously to ensure thorough mixing, followed by incubation at room temperature for 5 min and centrifugation at 12,000 × g for 15 min at 4°C. The aqueous phase was carefully transferred to a fresh centrifuge tube, and 500 *μ*L of isopropanol was added to precipitate the RNA. The mixture was allowed to stand at room temperature for 10 min and then centrifuged at 12,000 × g for 10 min at 4°C to pellet the RNA. The pellet was washed with 500 *μ*L of 75% ethanol and centrifuged at 7500 × g for 5 min at 4°C. The supernatant was discarded, and the pellet was retained for subsequent use. Finally, the RNA pellet was resuspended in 20 *μ*L of enzyme‐free water, mixed thoroughly, and stored overnight at 4°C. RNA concentration measurement, reverse transcription, and PCR reactions were performed as previously described in detail [17].

### 2.8. Statistical Analyses

Statistical analyses were performed using R software (v 4.2.0). Group comparisons between two independent samples were assessed with two‐tailed *t*‐tests if the data were approximately normally distributed; if normality was in question, a nonparametric test (Mann–Whitney *U*) was considered. Correlations between continuous variables were evaluated with Pearson′s correlation coefficient when both variables were approximately normally distributed, and with Spearman′s rank correlation when at least one variable was ordinal or not normally distributed. The two‐sided test with *p* < 0.05 was considered statistically significant. For downstream exploratory analyses, including immune infiltration correlations, disease‐gene correlations, and pathway enrichment scans, formal multiple‐testing correction was not uniformly applied. These analyses should be interpreted as hypothesis‐generating, and the findings warrant confirmatory validation in independent cohorts. For the clinical RT‐qPCR validation cohort, group comparisons were performed to evaluate whether peripheral‐blood expression of candidate genes differed between DR patients and healthy controls. Because the control group consisted of nondiabetic healthy individuals rather than diabetic patients without retinopathy, diabetes duration was not defined in controls. Therefore, the validation cohort was not designed to estimate the HbA1c‐ or diabetes duration–independent association between *ZFP36L1* expression and DR status. The clinical validation analysis should be interpreted as supportive expression validation rather than as a covariate‐adjusted etiological analysis.

## 3. Results

**Figure 1:**
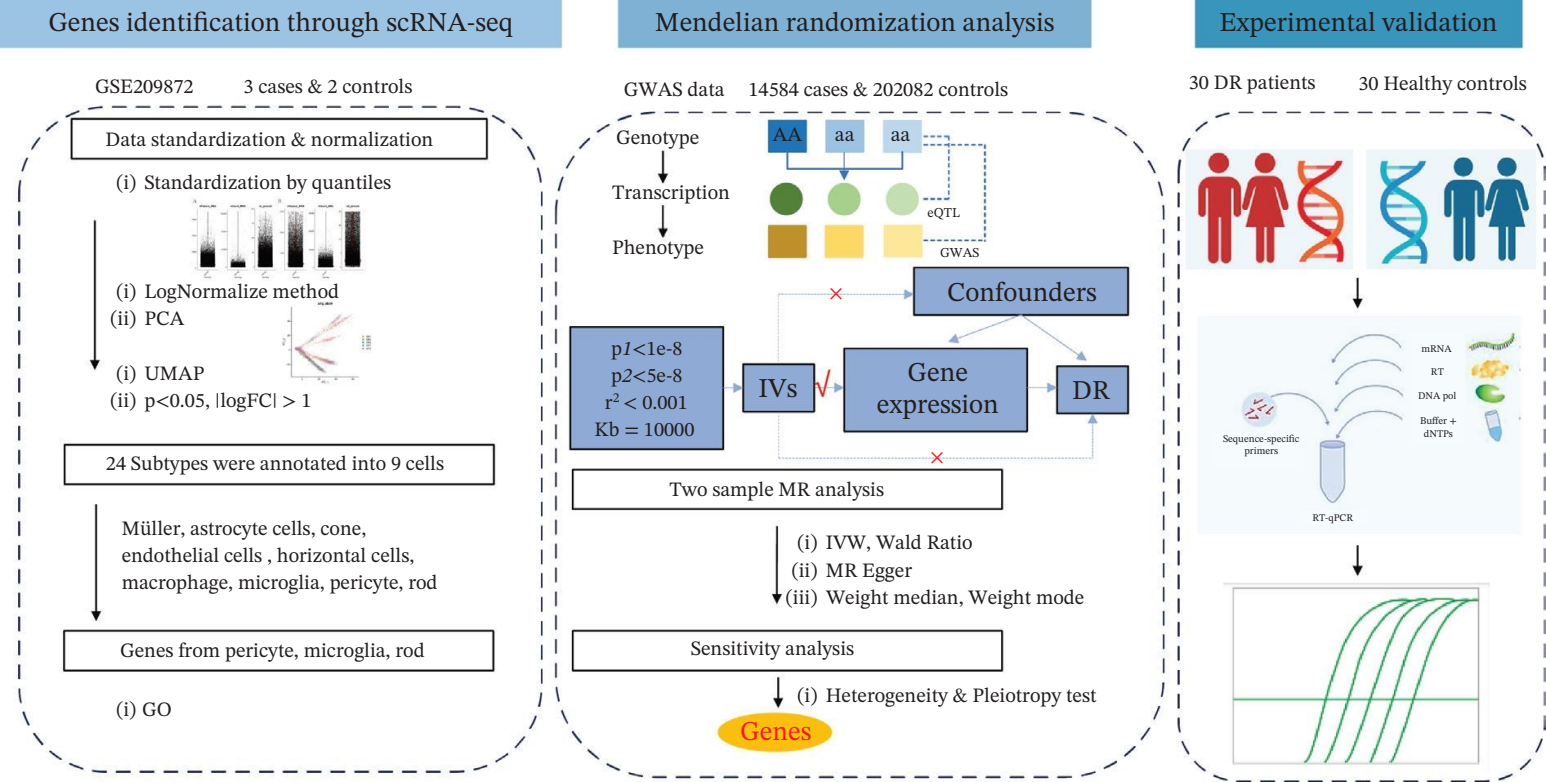
Flowchart of the study design. PCA, principal component analysis; UMAP, uniform manifold approximation and projection; IVs, instrumental variables; IVW, inverse‐variance weighted.

The overall study design is illustrated in Figure 1.

### 3.1. Subtype Annotation and Functional Enrichment Analysis

**Figure 2:**
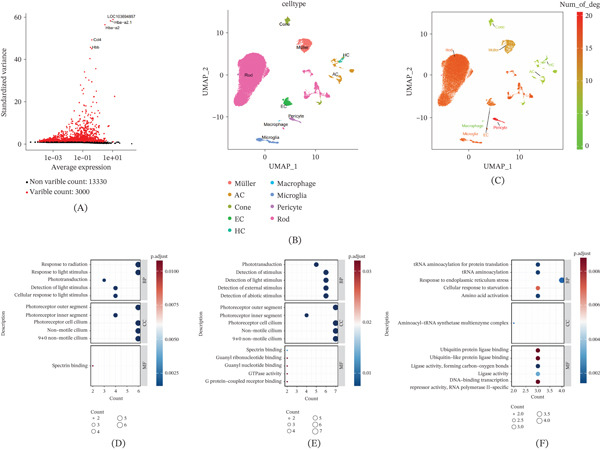
(A) The Top 5 genes exhibiting the highest standardized variance. (B) UMAP plot showing different cell types. Cells assigned to the same cluster are similarly colored. AC, amacrine cell; EC, endothelial cell; HC, horizontal cell. (C) The feature plot shows the number of DEGs in different cells under DR and normal conditions. The three cells with the largest numbers were pericytes, microglia, and rod. (D–F) Gene Ontology enrichment analysis of (D) pericytes, (E) microglia, and (F) rod cells. The enriched terms are categorized into biological process (BP), cellular component (CC), and molecular function (MF). Dot size represents the number of enriched genes (count), and color gradient indicates the adjusted *p* value.

Following data retrieval, samples were filtered based on nFeature RNA and percent.mt criteria, resulting in a refined dataset. Expression patterns of genes within samples were visualized, with the top 5 genes exhibiting the highest standardized variance marked for emphasis (Figure 2A). These genes represent transcriptional signatures associated with nonneuronal cell populations in the retina, including vascular and immune cells, thereby reflecting the cellular heterogeneity captured by the single‐cell dataset GSE209872. Importantly, none of these top variable genes showed a diabetes‐specific expression pattern or significant association with DR in our analysis—hence, they were not the focus of downstream experiments. Cells were subsequently grouped into 24 clusters using UMAP analysis, and the 24 identified subtypes were annotated into 9 cell categories: Müller, amacrine cells, cone, endothelial cells, horizontal cells, macrophage, microglia, pericyte, and rod cells (Figure 2B). Furthermore, profiled DEGs across major retinal cell types isolated from various retinal cells in normal rats and diabetic rats revealed that pericytes, microglia, and rod changed significantly in the cases (Figure 2C). GO enrichment analysis showed that pericytes and microglia were related to the detection of light stimulus, and previous studies have shown that pericyte deletion is one of the mechanisms of DR (Figure 2D–F) [18]. Given the limited sample size of the scRNA‐seq dataset, these single‐cell findings should be regarded as exploratory and hypothesis‐generating rather than definitive evidence of cell‐type‐specific causality.

### 3.2. MR Analysis Identifies Eight Candidate Genes Including *ZFP36L1*

We performed two‐sample MR using blood eQTL data as the exposure (expression quantitative trait loci from peripheral blood) and a DR GWAS as the outcome. Although this analysis identified several candidate genes, we note that the tissue context is a limitation. This analysis identified eight genes, *CA2, CTSH, GLUL, IER3, LPCAT1, MXI1, PDGFRB, ZFP36L1*, associated with a lower risk, *CA2* (OR = 0.944, 95% confidence intervals [CI] 0.904−0.986, *p* = 0.010), *LPCAT1* (OR = 0.871, 95% CI = 0.769−0.987, *p* = 0.030), *MXI1* (OR = 0.923, 95% CI = 0.863−0.987, *p* = 0.019), *PDGFRB* (OR = 0.818, 95% CI = 0.714−0.937,*p* = 0.004), or a higher risk, *CTSH* (OR = 1.069, 95% CI = 1.028−1.111, *p* = 0.001), *GLUL* (OR = 1.127, 95% CI = 1.013−1.254, *p* = 0.027), *ZFP36L1* (OR = 1.156, 95% CI = 1.054−1.269, *p* = 0.002), *IER3* (OR = 1.211, 95% CI = 1.090−1.347, *p* < 0.001).

**Figure 3:**
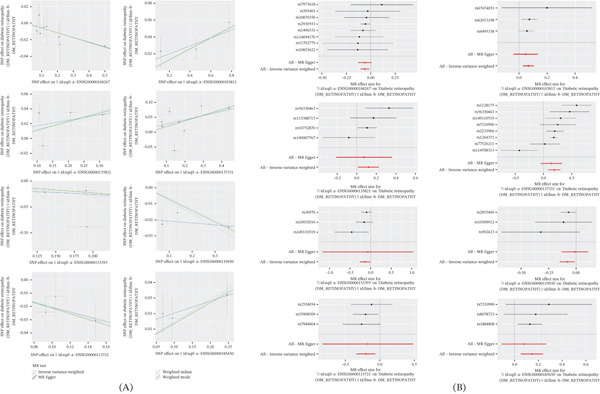
(A) Scatter plots of the SNP effects. One SNP that is utilized as an instrumental variable is shown by each dot. All SNPs′ effects on genes are plotted on the *x*‐axis. The estimated causal effect for each SNP on DR is shown on the *y*‐axis. (B) Forest plots of causal effects. The estimated causal effect of all SNPs on the outcome is represented by red points. The line segments indicate 95% confidence intervals, and the position of each dot reflects the magnitude of the causal effect.

**Figure 4:**
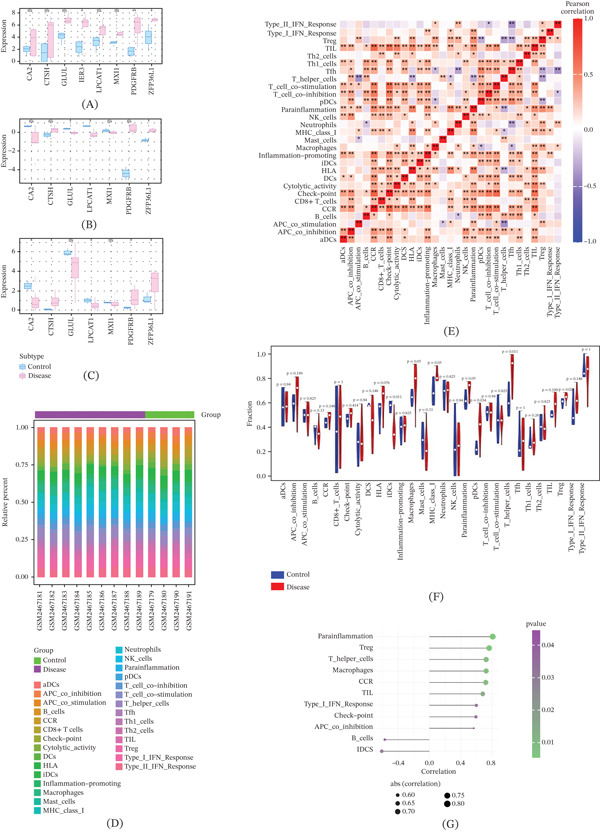
(A) Validation of *ZFP36L1* using dataset GSE94019. (B) Validation of *ZFP36L1* using dataset GSE60436. (C) Validation of *ZFP36L1* using dataset GSE102485. (D) Percentage of immune cells between the normal group and the DR patients. (E) Interaction analysis among 29 different immune cells in DR patients ( ^∗^ represents *p* < 0.05,  ^∗∗^ represents *p* < 0.01, and  ^∗∗∗^ represents *p* < 0.001). (F) Comparisons of immune cells between the normal control group and DR group. (G) Correlations between *ZFP36L1* and immune infiltration cells.

Significant heterogeneity was detected for *IER3* (Cochran′s *Q*, *p* < 0.05), prompting us to re‐estimate its causal effect with a multiplicative random‐effects IVW model. Detailed instrument characteristics and sensitivity metrics, including the number of SNPs, SNP lists, IVW results, MR‐Egger intercept statistics, and Cochran′s *Q* statistics, are provided in Table S1. Results in Table S1 demonstrate a significant positive causal relationship between elevated *IER3* expression and increased DR risk (*p* < 0.001). The eQTL of eight genes showed causal relationships with DR (Figure 3A,B). Funnel plots are additionally provided to assess potential small‐study effects and directional pleiotropy across the instrumental SNPs (Figure S1). Leave‐one‐out sensitivity analysis was conducted to assess the reliability of the causal relationships among the eight genes. Results showed that removing any single SNP had no significant impact on the overall causal estimate, indicating that the eight causal relationships are robust. Transcriptional validation using the dataset GSE94019, GSE60436, and GSE102485 highlighted significant expression differences for *ZFP36L1* (Figure 4A–C). Instruments for each gene‐expression exposure were selected at genome‐wide significance (*p* < 1 × 10^−8^) and LD‐pruned to near‐independence (*R* ^2^ clumping) prior to harmonization to minimize weak‐instrument bias; SNP‐level *F*‐statistics and variance explained (*R* ^2^) could not be uniformly derived from the available summary eQTL outputs.

Among the eight MR‐identified genes, *ZFP36L1* was prioritized for downstream analysis based on convergent evidence from two complementary layers: (1) a significant causal association with DR risk in MR analysis (OR = 1.156, *p* = 0.002) and (2) consistent upregulation across three independent external transcriptomic datasets (GSE94019, GSE60436, and GSE102485). Notably, *ZFP36L1* was the only gene among the eight that showed reproducible expression differences in all three validation datasets, providing a strong rationale for focused downstream investigation.

### 3.3. Alterations in the Retinal Immune Microenvironment

**Figure 5:**
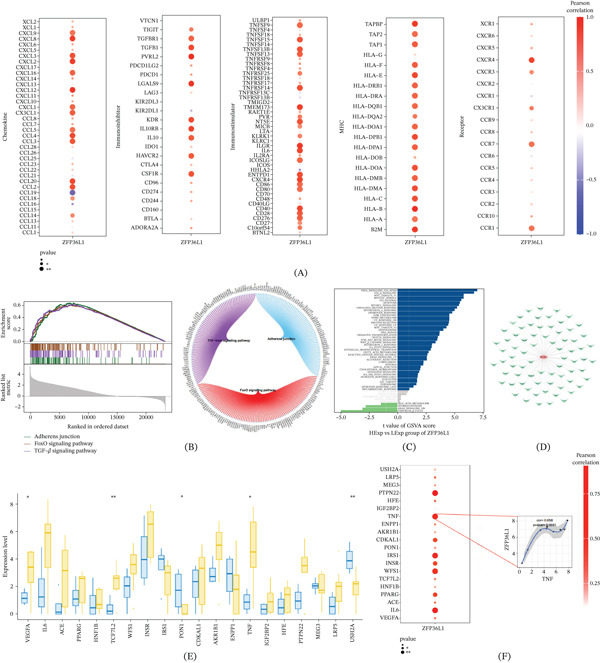
(A) Correlations between *ZFP36L1* and different immune factors. (B, C) GSVA and GSEA analysis of high and low expression of *ZFP36L1*. (D) Transcriptional regulatory network of *ZFP36L1*. (E) The comparisons of the expression of multiple disease‐related genes between the control and DR patients. (F) Bubble map for the Pearson correlations between *ZFP36L1* and disease‐related genes. (The bigger the circle, the closer the *p* value was to zero; the redder the color, the stronger the positive correlation; the deeper of the purple color, the stronger the negative correlation).

We further explored the potential molecular mechanisms by which key genes influence the progression of DR by analyzing the relationship between key genes and immune infiltration in the DR dataset, and this study demonstrated the percentage of immune cell content in each patient as well as the correlation between immune cell (Figure 4D,E). Patients in the disease group exhibited higher levels of plasma dendritic cells, T helper cells, and regulatory T cells (Tregs) compared with normal individuals (Figure 4F). *ZFP36L1* exhibited positive correlations with parainflammation, Tregs, T helper cells, and macrophages, and negative correlations with immature dendritic cells and B cells (Figure 4G). These observations are correlational and derived from transcriptome‐based immune inference rather than direct immunophenotyping. As with other downstream correlation‐based analyses in this study, formal multiple‐testing correction was not applied, and these findings should be regarded as exploratory. Using the TISIDB resource, we obtained correlations between *ZFP36L1* and different immune factors including immunosuppressive factors, immunostimulatory factors, chemokines, and receptors. The resulting associations indicate that the key gene expression correlates with infiltrating immune abundance(Figure 5A).

### 3.4. *ZFP36L1* Enrichment in Inflammatory and Angiogenic Pathways

To dissect how the key gene drives disease evolution, we conducted enrichment analyses of the signaling pathways involved in these key genes. The results from GSEA showed that *ZFP36L1* was enriched in pathways such as the adherens junction, the FoxO signaling pathway, and the TGF‐*β* signaling pathway (Figure 5B). GSVA revealed that high expression of *ZFP36L1* was enriched in signaling pathways such as TNFA‐α signaling via NF‐*κ*B, TGF‐*β* signaling, and the p53 pathway (Figure 5C). These findings suggest that key genes may modulate DR progression through the above routes.

### 3.5. Regulatory Network of *ZFP36L1* in Single‐Cell Data

By submitting *ZFP36L1* as the query gene set to Cistrome DB, we retrieved 74 high‐confidence transcription factors after stringent filtering. The resulting transcription factor–target interactions were imported into Cytoscape to generate an integrated regulatory map centered on the core DR genes (Figure 5D).

### 3.6. Disease‐Regulating Genes and Correlation Analysis

We analyzed the expression levels of the 20 genes with the top relevance score, analyzed the intergroup expression differences of disease genes, and found that *VEGFA, TCF7L2, PON1, TNF*, and *USH2A* expression was significantly different between the two groups of patients (Figure 5E). Next, we performed correlation analysis of *ZFP36L1* and DR regulatory genes (Figure 5F), and found that the expression levels of key gene and disease‐related genes were significantly correlated, with *ZFP36L1* being significantly positively correlated with TNF (*r* = 0.858, *p* = 0.003).

### 3.7. Single‐Cell Expression Analysis and Pathway Correlation

**Figure 6:**
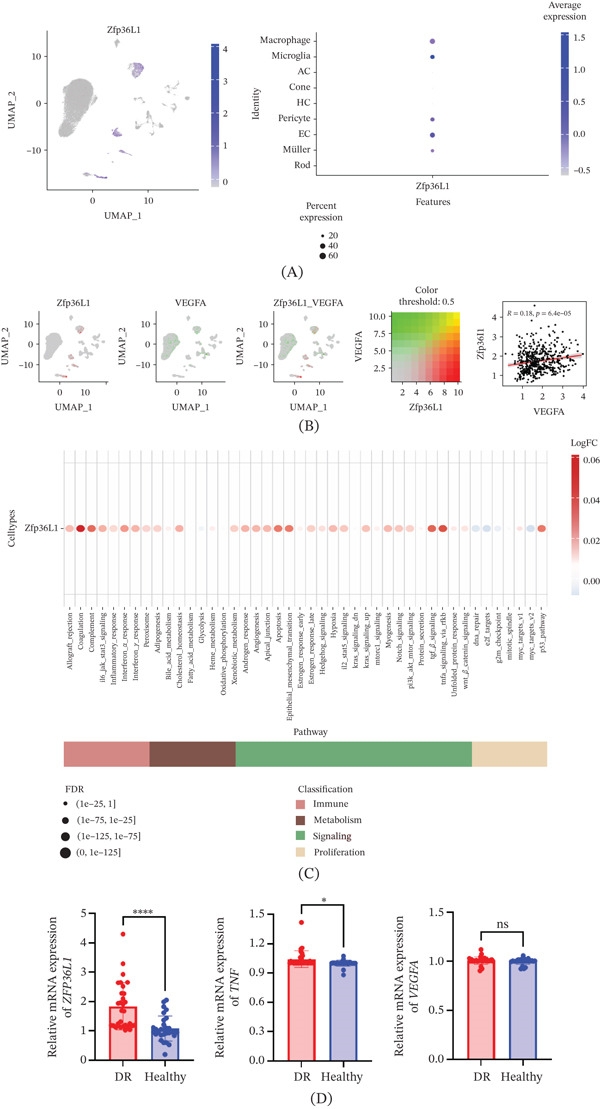
(A) Distribution of *ZFP36L1* in single‐cell. (B) *ZFP36L1* and *VEGFA*′s similar expression patterns and relationships. (C) Single‐cell gene set enrichment analysis. (D) Expression of characteristic genes in peripheral blood of healthy individuals and DR patients. *N* = 60, ns represents *p* > 0.05,  ^∗^ represents *p* < 0.05, and  ^∗∗∗∗^ represents *p* < 0.0001.

Single‐cell analysis of key gene expression across various cell types (Müller, amacrine cell, cone, endothelial cell, horizontal cell, macrophage, microglia, pericyte, and rod) demonstrated their differential expression patterns (Figure 6A). Coexpression analysis highlighted shared expression patterns between disease‐regulating gene (*VEGFA*) and *ZFP36L1*, along with their correlation (*r* = 0.180, *p* < 0.001) (Figure 6B). Finally, immune and metabolic pathways were quantitatively assessed using the AUCell algorithm, and the correlations between *ZFP36L1* and these pathways were systematically analyzed and presented (Figure 6C).

### 3.8. Experimental Validation

To provide supportive clinical expression validation of the bioinformatic findings, we performed RT‐qPCR to quantify *ZFP36L1*, *TNF*, and *VEGFA* expression in human peripheral blood. In the cohort, the DR group included 9/30 men (30.0%) and the control group included 10/30 men (33.3%) (*p* = 0.781). The mean age was 56.9 ± 13.7 years in the DR group and 54.9 ± 11.0 years in the control group (*p* = 0.430). HbA1c was 10.6*%* ± 2.3*%* in the DR group and 5.1*%* ± 0.2*%* in the control group (*p* < 0.001). The median diabetes duration in the DR group was 120 (69–180) months, and diabetic kidney disease was present in 23/30 DR patients (76.7%). *ZFP36L1* (fold change ratio: 1.707, 95% CI: 1.367–2.131; *p* < 0.0001) and *TNF* (fold change ratio: 1.039, 95% CI: 1.009–1.070; *p* = 0.011) were expressed at higher levels in DR patients than in healthy controls. In contrast, *VEGFA* did not differ significantly between groups and did not meet our prespecified criterion for a trend (fold change ratio: 1.008, 95% CI: 0.990–1.026; *p* = 0.364) (Figure 6D). Given the small sample size and three markers tested, we treated this as an exploratory analysis without formal correction for multiple comparisons. The present validation cohort was not suitable for deriving a biologically interpretable covariate‐adjusted estimate of the independent association between DR status and *ZFP36L1* expression. This limitation was mainly due to the structure of the comparison group rather than sample size alone. Specifically, the control group consisted of nondiabetic healthy individuals, diabetes duration was not defined in controls, and HbA1c values showed substantial between‐group differences with limited clinical overlap. Therefore, the RT‐qPCR findings should be interpreted as supportive evidence of increased peripheral‐blood *ZFP36L1* expression in DR patients compared with healthy controls, rather than as proof that this increase is independent of glycemic control, diabetes duration, or diabetes‐related systemic factors. A larger, well‐characterized patient cohort will be needed to confirm these findings, allow subgroup analyses (diabetic patients without DR vs. nonproliferative DR vs. proliferative DR), and adjust for potential confounders.

## 4. Discussion

Our study prioritizes *ZFP36L1* as a candidate susceptibility gene associated with DR risk through integrative multiomics analysis and supportive peripheral‐blood expression validation. Key observations include (1) genetic evidence from blood eQTL‐based MR supporting an association between genetically predicted *ZFP36L1* expression and DR risk and (2) pathway enrichment analyses suggesting potential links between *ZFP36L1* and inflammatory or angiogenic pathways, including NF‐*κ*B and TGF‐*β* signaling. These findings suggest that *ZFP36L1* may be involved in biological processes relevant to DR, although its retina‐specific function and therapeutic relevance remain to be established experimentally. Our MR analysis leveraged whole‐blood eQTLs rather than retina‐specific eQTLs. Therefore, the identified association should be interpreted primarily as evidence that systemic genetic regulation of *ZFP36L1* is associated with DR risk, rather than as proof of a retina‐autonomous mechanism.

*ZFP36L1*, also known as *TIS11b* or *BRF1*, is a zinc‐finger RNA–binding protein that mediates mRNA decay by recognizing conserved adenylate‐uridylate‐rich elements (AREs) located in the 3 ^′^ untranslated regions of target transcripts [19]. As a post‐transcriptional regulator widely expressed in eukaryotes, *ZFP36L1* is involved in the modification, transportation, localization, degradation, and translation of mRNAs [20]. In recent years, aberrant expression of *ZFP36L1* has been increasingly implicated in various malignancies. Additionally, its critical role in nonneoplastic diseases, including metabolic disorders, immune responses, and diseases associated with angiogenesis, has progressively drawn attention. Recently, GWAS have revealed it is significantly associated with type 1 diabetes [21].

Through an integrative approach combining scRNA‐seq and MR analysis, we identified *ZFP36L1* as a novel potential susceptibility gene for DR. Furthermore, peripheral‐blood RT‐qPCR validation showed higher *ZFP36L1* expression in DR patients than in healthy controls. However, the clinical validation results should be interpreted with caution. The validation cohort compared DR patients with nondiabetic healthy controls, rather than with diabetic patients without retinopathy. Therefore, the observed increase in peripheral‐blood *ZFP36L1* expression cannot be attributed specifically to retinopathy independent of diabetes, glycemic control, diabetes duration, or diabetes‐related systemic inflammation. Although a limited multivariable model could be fitted numerically, diabetes duration is not biologically defined in healthy controls, and HbA1c values showed limited overlap between the two groups. Under these conditions, covariate‐adjusted estimates would be highly dependent on model extrapolation and would be difficult to interpret biologically. Future studies including diabetic non‐DR controls will be necessary to determine whether ZFP36L1 is independently associated with DR beyond the effects of chronic hyperglycemia and diabetes itself. We next explored the underlying mechanism. Prolonged hyperglycemia triggers pathological processes—including inflammation, angiogenesis, and pericyte loss—that collectively contribute to DR progression [22]. Previous studies have revealed the correlation of *ZFP36L1* in inflammation and angiogenesis, two key pathological mechanisms underlying DR [19, 23].

Studies have shown that the inflammatory response not only plays a driving role in the development of DR, but also involves interactions between multiple cytokines, chemokines, and adhesion molecules, forming a complex molecular network that drives the inflammatory and pathological cascade of DR [18, 24, 25]. Our study indicates that *ZFP36L1* is positively correlated with parainflammation, Tregs, T helper cells, and macrophages, and negatively correlated with B cells, and positively correlated with the expression of TNF‐*α* and IL‐6, in accordance with the reported roles of *ZFP36L1* in immune responses [21, 26]. One potential explanation for observing higher Treg signals in DR is a compensatory response: Tregs may be mobilized in an attempt to counteract the heightened inflammation. However, these Tregs could be functionally impaired or insufficient in the diabetic milieu, meaning inflammation still prevails [8]. Notably, DR immunity is most consistently linked to microglia/macrophage activation, whereas evidence for adaptive T cells (including Tregs) remains limited and debated [27]. In DR, innate immune activation—especially involving microglia and macrophages—has been more consistently supported than adaptive T‐cell infiltration, and transcriptome‐based immune deconvolution cannot by itself establish the identity, abundance, or function of retinal immune populations. We therefore regard these findings as hypothesis‐generating and in need of orthogonal validation, such as immunostaining, flow cytometry, or immune‐focused single‐cell/spatial profiling. The proinflammatory cytokine TNF‐*α* is secreted by T cells, natural killer cells, or macrophages and contributes significantly to DR. Elevated TNF‐*α* levels have been observed in the serum, vitreous, and aqueous humor of patients with DR, with serum levels showing a positive correlation with disease severity [28–30]. IL‐6 is a pleiotropic inflammatory mediator that modulates immune responses, increases vascular permeability, and promotes angiogenesis [31]. We found that *ZFP36L1* was enriched in the NF‐*κ*B signaling pathway. NF‐*κ*B activation is implicated in inflammatory and apoptotic pathways, where it modulates the expression of inflammatory genes and accelerates apoptosis and neovascularization in DR [32]. Collectively, these data suggest that *ZFP36L1* participates in DR progression by modulating inflammatory responses and immune functions, potentially involving the NF‐*κ*B signaling pathway. Additionally, the mechanisms by which *ZFP36L1*‐expressing cells may influence immune cell recruitment or activation in the retina remain to be explored. We did not perform an explicit cell–cell communication analysis in this study; such analyses, including ligand–receptor interaction mapping, will be important in future work to determine how *ZFP36L1*‐high retinal cells could recruit or regulate immune subsets. This remains a priority for further investigation. Our MR analysis leveraged eQTLs from whole blood rather than retinal tissue. This tissue mismatch implies that the identified genetic association reflects gene regulation in blood. The observed causal link for *ZFP36L1* may therefore reflect a systemic inflammatory effect on DR risk rather than a direct retinal action. We acknowledge that using blood cis‐eQTLs as instruments may reduce validity if the gene′s effect is tissue‐specific. Unfortunately, large‐scale retina eQTL datasets are not yet available; we therefore used the best available data (blood eQTLGen). We explicitly acknowledge that the MR findings should be interpreted with this tissue context in mind.

Our study confirms *VEGFA* upregulation in DR, consistent with the established hypoxia–VEGF–neovascularization pathogenic cycle [33]. Surprisingly, we observed a positive correlation between *ZFP36L1* and *VEGFA* expression (*p* < 0.001), contrasting with *ZFP36L1*′s known role as a VEGF mRNA destabilizer [34]. This apparent contradiction may reflect context‐dependent functions of *ZFP36L1*, analogous to its dual roles in cancer biology, where it exhibits both tumor‐suppressive and oncogenic properties depending on tumor type and stage [35]. Importantly, our pathway analyses revealed enrichment of *ZFP36L1* in TGF‐*β* signaling, providing a plausible mechanistic link: TGF‐*β* is not only a potent inducer of *ZFP36L1* expression but also a known driver of VEGF‐mediated angiogenesis in DR [36–39]. Hypoxia may further amplify this relationship by enhancing *ZFP36L1* mRNA stability [40]. We propose that these findings suggest a potential feedforward loop in DR pathogenesis, in which TGF‐*β* activation induces both *ZFP36L1* and VEGF, whereas hypoxic conditions stabilize *ZFP36L1* expression, thereby exacerbating angiogenic signaling. This model reconciles our unexpected findings with established literature while highlighting the complex, context‐dependent regulation of *ZFP36L1* in vascular pathophysiology [41]. The proposed relationship between *ZFP36L1* and angiogenic signaling should be considered speculative at present. Functional experiments, such as *ZFP36L1* knockdown/overexpression in disease‐relevant retinal cell types and assessment of VEGFA‐related downstream effects, will be necessary to test this model directly. The validation cohort was not designed for robust covariate‐adjusted inference or stage‐stratified analysis, primarily because it lacked diabetic non‐DR controls and detailed DR staging information. Therefore, the clinical validation data should be regarded as supportive rather than definitive.

*ZFP36L1* functions as an RNA‐binding protein that binds AREs in target mRNAs, thereby promoting mRNA turnover and decay [42]. Many inflammatory and angiogenic genes implicated in DR contain AREs. For example, TNF‐*α* and ICAM‐1 are well‐known examples of inflammatory transcripts subject to ARE‐associated posttranscriptional regulation, and CCL2/MCP‐1 is also regulated via functional 3 ^′^UTR motifs that influence RNA‐binding protein interactions and mRNA stability [43–45]. *ZFP36L1* may modulate DR pathology by regulating such transcripts. If *ZFP36L1* is upregulated, it could reduce the stability of proinflammatory mRNAs, potentially acting as a protective mechanism. However, in the diabetic retina, chronic stress signals—such as hyperglycemia and cytokines—may alter *ZFP36L1′s* effectiveness or expression dynamics. Therefore, investigating *ZFP36L1′s* interaction with ARE‐containing mRNAs (e.g., ICAM‐1 and MCP‐1) in retinal cells could be an exploratory analysis to further elucidate its role in DR.

Clinically, whether *ZFP36L1* expression levels in blood or ocular fluids correlate with DR severity or patient outcomes remains to be determined. Importantly, the present validation cohort cannot distinguish DR‐specific expression changes from those related to diabetes, hyperglycemia, or systemic inflammatory status because diabetic patients without DR were not included. Our current cohort was not powered to detect associations with specific endpoints such as progression from NPDR to PDR, changes in visual acuity, or treatment responses. Future studies in larger patient populations should evaluate whether *ZFP36L1* could serve as a biomarker for disease stage or prognosis. We also address the potential of *ZFP36L1* as a therapeutic target. *ZFP36L1* is an RNA‐binding protein that promotes mRNA decay, including that of inflammatory factors [46]. Modulating its activity may confer anti‐inflammatory or antiangiogenic benefits in DR. However, directly targeting RNA‐binding proteins with small molecules remains challenging. Any therapeutic strategy would likely need to modulate *ZFP36L1* indirectly—for instance, by pathways that regulate its expression or by mimicking its mRNA‐destabilizing effects. Although *ZFP36L1* holds promise, substantial work remains to evaluate its druggability and safety.

Recently, several studies have also employed multiomics analyses to explore potential key genes in DR, providing a basis for guiding the prevention and treatment of DR [14–16]. The primary strengths of our study lie in combining scRNA‐seq with population‐based studies using MR integration of gene expression data and GWAS, enabling investigation of underlying genes and mechanisms in DR, alongside refined immune infiltration analyses and experimental validation. MR validated the potential causal role of *ZFP36L1* in the DR pathophysiology by treating the linked eQTL as an instrumental variable, an inference further substantiated by confirmatory experiments in human participants. These convergent lines of evidence position *ZFP36L1* as a potential therapeutic node that simultaneously modulates inflammation and angiogenesis.

Although this study provides novel insights into *ZFP36L1*′s role in DR, several limitations should be acknowledged. First, the scRNA‐seq analysis was based on a public rat diabetic/DR dataset with a small cohort (three DR vs. two controls), limiting statistical power and generalizability. In addition, NPDR/PDR clinical staging is not applicable to this rat model; the samples are defined by timepoints (2/4/8 weeks) after diabetes induction, precluding clinical stage–stratified single‐cell comparisons. Therefore, diabetes duration could not be modeled as a covariate and robust time‐point trend analyses were not feasible. Therefore, the single‐cell results should be interpreted as exploratory and hypothesis‐generating, and larger single‐cell datasets—ideally including human cohorts with defined clinical stages—are needed for a comprehensive progression atlas. Second, although we observed higher *ZFP36L1* expression in peripheral blood from DR patients than in healthy controls, the clinical validation cohort did not include diabetic patients without DR. Therefore, we cannot determine whether increased *ZFP36L1* expression reflects DR itself, chronic hyperglycemia, diabetes duration, diabetes‐related systemic inflammation, or their combined effects. However, obtaining retinal biopsies from patients is not feasible due to ethical and technical constraints; therefore, blood was used as a surrogate tissue in this study. We acknowledge that gene expression in blood may partly reflect systemic inflammation, glycemic exposure, or other diabetes‐related systemic processes rather than retinal‐specific pathology. Furthermore, our study did not include functional perturbation experiments, and the detailed NPDR/PDR staging data were not available for the validation cohort, precluding stage‐stratified analysis of *ZFP36L1* expression. Thus, although we identified associations and potential causal links, we have not yet demonstrated that directly modifying *ZFP36L1* expression can alter DR phenotypes. This lack of in vitro or in vivo functional validation is a limitation of the current work. Ideally, retina‐specific eQTL data or ocular samples would be used to validate genetic findings. In the absence of large retina eQTL resources, our MR analysis was based on blood eQTLs, an approach that has been extensively utilized in ophthalmological MR studies [47]. Third, our genetic findings were derived from predominantly European ancestry datasets (GWAS and eQTL), whereas our clinical validation was conducted in a Chinese population. This ancestry discordance could affect the applicability of the results. We therefore caution that our findings should be validated in multiethnic cohorts. Mechanistically, although we identified *ZFP36L1*′s associations with TNF‐*α* and VEGF pathways, its specific RNA targets and posttranscriptional regulatory networks require experimental mapping. The inferred immune shifts (e.g., increased Tregs) are observational and derived from bulk‐retina deconvolution, where cell‐type mixing can dilute immune signals and bias proportion estimates. Our immune infiltration analysis was limited to broad immune cell categories (e.g., total T helper cells) due to sample size constraints. We did not further subdivide immune subtypes (e.g., Th1 vs. Th2 vs. Th17 or specific macrophage subsets) as the small cell numbers precluded reliable distinction. These limitations highlight opportunities for future work: Larger single‐cell atlases, retinal *ZFP36L1* perturbation models, and longitudinal clinical cohorts including diabetic patients without DR would further clarify whether *ZFP36L1* is specifically associated with DR onset, severity, or progression independent of glycemic control and diabetes duration.

## 5. Conclusion

In summary, our integrative analysis prioritized *ZFP36L1* as a candidate susceptibility gene associated with DR risk. Blood eQTL‐based MR suggested that genetically regulated *ZFP36L1* expression is associated with DR risk, and peripheral blood RT‐qPCR showed increased *ZFP36L1* expression in DR patients compared with healthy controls. Retinal single‐cell and pathway analyses provided supportive, hypothesis‐generating evidence that *ZFP36L1* may be linked to inflammatory and angiogenic pathways relevant to DR. However, these findings do not establish a retina‐specific regulatory mechanism. Further studies using retina‐specific datasets and functional perturbation experiments are required to determine whether *ZFP36L1* directly regulates retinal inflammation or angiogenesis and whether it has diagnostic or therapeutic potential.

**Nomenclature**
AUCell: area under the curve cell
CI: confidence interval
DEG: differentially expressed gene
DR: diabetic retinopathy
eQTL: expression quantitative trait locus
GEO: Gene Expression Omnibus
GO: Gene Ontology
GSEA: gene set enrichment analysis
GSVA: gene set variation analysis
GWAS: genome‐wide association study
IL: interleukin
IV: instrumental variable
IVW: inverse‐variance weighted
LD: linkage disequilibrium
MR: Mendelian randomization
NF‐*κ*B: nuclear factor kappa‐B
NPDR: nonproliferative diabetic retinopathy
OR: odds ratio
PCA: principal component analysis
PDR: proliferative diabetic retinopathy
RT‐qPCR: reverse transcription quantitative polymerase chain reaction
scRNA‐seq: single‐cell RNA sequencing
SNP: single‐nucleotide polymorphism
TGF‐*β*
: transforming growth factor beta
TNF: tumor necrosis factor
Tregs: regulatory T cells
UMAP: uniform manifold approximation and projection
VEGF: vascular endothelial growth factor

## Author Contributions

Conceptualization: Wei Qiang; data curation: Chenwen Luo; funding acquisition: Mingqian He, Bingyin Shi, and Wei Qiang; investigation: Shengnan Chen; project administration: Ruiqing Dong; resources: Ning Gao, Xiaoli Hui, and Mingqian He; supervision: Bingyin Shi; validation: Ruiqing Dong; writing—original draft: Ruiqing Dong and Xiaoli Hui; writing—review and editing: Ning Gao, Bingyin Shi, and Wei Qiang. Ruiqing Dong and Xiaoli Hui have contributed to the work equally and should be regarded as co‐first authors.

## Funding

This study was supported by the Key Research and Development Program of Shaanxi Province (2023‐ZDLSF‐40, 2021LL‐JB‐06) and the Natural Science Foundation Program of Shaanxi (2024JC‐YBQN‐0828).

## Disclosure

All authors have reviewed and approved the final submission of the manuscript.

## Ethics Statement

This study was conducted with approval from the Ethics Committee of the First Affiliated Hospital of Xi′an Jiaotong University (Approval No. XJTU1AF2023LSK–458). This study was conducted in accordance with the Declaration of Helsinki. Written informed consent was obtained from all participants. To ensure participant confidentiality, all patient data were anonymized prior to analysis, with personal identifiers removed from the dataset.

## Conflicts of Interest

The authors declare no conflicts of interest.

## Supporting Information

Additional supporting information can be found online in the Supporting Information section.

## Supporting information


**Supporting Information 1** Table S1: Instrument characteristics and sensitivity metrics for Mendelian randomization analyses of the eight genes associated with diabetic retinopathy.

**Supporting Information 2** Figure S1: Funnel plots for MR analyses of genetically predicted gene expression and diabetic retinopathy. Panels (A–H) correspond to (A) *CA2*, (B) *CTSH*, (C) *GLUL*, (D) *IER3*, (E) *LPCAT1*, (F) *MXI1*, (G) *PDGFRB*, and (H) *ZFP36L1*.

## Data Availability

All data analyzed during this study are included in the websites mentioned above.

## References

[1] Cheung N. , Mitchell P. , and Wong T. Y. , Diabetic Retinopathy, Lancet. (2010) 376, no. 9735, 124–136, 10.1016/S0140-6736(09)62124-3. 20580421

[2] Skol A. D. , Jung S. C. , Sokovic A. M. , Chen S. , Fazal S. , Sosina O. , Borkar P. P. , Lin A. , Sverdlov M. , Cao D. , Swaroop A. , Bebu I. , DCCT/EDIC Study group , Stranger B. E. , and Grassi M. A. , Integration of Genomics and Transcriptomics Predicts Diabetic Retinopathy Susceptibility Genes, Elife. (2020) 9, e59980, 10.7554/eLife.59980, 33164750. 33164750 PMC7728435

[3] Jampol L. M. , Glassman A. R. , and Sun J. , Evaluation and Care of Patients With Diabetic Retinopathy, New England Journal of Medicine. (2020) 382, no. 17, 1629–1637, 10.1056/NEJMra1909637. 32320570

[4] Hutton D. W. , Glassman A. R. , Liu D. , Sun J. K. , DRCR Retina Network , Sneath M. , Chen M. , Jelemensky P. A. , Miller R. , Basham S. R. , Raphael T. L. , Harara A. M. , Berger B. B. , Jhaveri C. D. , Stovall C. C. , Renfroe C. , Vega Pereira D. , Wilson D. M. , Makkouk F. , Jonna G. , Gunderson I. , Chexal S. , Gatavaski V. , Ren Y. , Irons A. N. , Rego B. , Weinberg D. V. , Dorsey E. , Nelson E. , Sheppard H. , McKenney K. C. , Chen N. , Wirostko W. J. , Ghuman A. T. , Arevalo A. , Petersen A. J. , Leslie A. H. , Sharma A. G. , Kiesel C. , Peters C. Y. , Knips E. , Walker J. P. , Mears K. A. , Maro K. , Toleman L. T. , Raskauskas P. A. , Kiesel R. K. , Meeks A. , Rhymes G. K. , Gardner G. R. , Shami M. , Saldivar Y. , Schlossman D. K. , Weimann E. S. , Rhee J. W. , Sun J. K. , Cavallerano J. D. , Tran K. V. , Bestourous L. , Stockman M. E. , Sehizadeh M. , Silva P. S. , Cavicchi R. W. , Shah S. T. , Papaconstantinou S. L. , Olesker T. , Murtha T. J. , Carli W. , Wilson A. H. , Antoszyk A. N. , Price A. K. , Gentile A. K. , Murphy B. A. , Shore C. A. , Mutch C. , Fleming C. J. , Browning D. , McClain D. , Allen J. B. , Clark J. , McShea K. T. , Bratcher K. A. , Jackson L. A. , Clark L. M. , Watson L. , Nayar M. D. , Punjabi O. S. , Ennis S. A. , Stobbe S. , Fredenberg S. L. , Ross T. A. , Balasubramaniam U. M. , Harless A. M. , Novak C. K. , Harris C. , Brown E. , Fiscus H. , White L. , Retrum M. K. , Maturi R. K. , Morrow S. J. , Saxe S. J. , Mahesh V. , Sarmiento Y. , Gilbreath C. , Griffone H. A. , Wheeler J. , Googe J. M. , Asher J. , Walsh J. , Milstead K. , Oliver K. , Anderson N. G. , Shuler R. K. , Lince R. E. , Oelrich S. M. , Perkins S. L. , Morris S. , McCoy S. , Seitz V. L. , al Moshmosh A. , Nicholson A. M. , Tucker A. Y. , Clow C. , Trichonas G. , Miranda G. R. , Baum-Rawraway I. , Randhawa J. K. , O′Malley J. , Wash L. M. , Richards L. A. , Petrosky M. N. , Allchin P. , Kurup S. K. , Pelton S. , Brewer A. N. , Hollingshead A. M. , Hughes A. , Cheadle M. , Thomas M. , Sohl R. , Kingsley R. M. , Burris R. , Almeida S. R. , Icks S. , Shah V. A. , Camp A. B. , Baker C. W. , Baker J. D. , Sedberry K. S. , Orr M. J. , Sharp M. J. , Kettler S. , Alcaraz S. L. , Caldwell T. M. , Listerman A. D. , Igoe A. L. , Feehan C. E. , McLouth D. S. , Kuitula D. E. , Truax E. , Garber F. W. , Cruz H. L. , Zheutlin J. D. , DeHorn K. U. , Glazer L. C. , Homann M. A. , Crown P. D. , Weatherbee S. , Hutson A. , Richter B. A. , Almanza B. A. , Wykoff C. C. , Henry C. R. , Garcia D. , Kegley E. N. , Vela M. , Fish R. H. , McCoy T. , Sneed V. A. , Alset A. E. , Sierpina D. I. , Easterly M. , Rauser M. E. , Tellez M. , Hernandez R. , Ramirez T. L. , Reddy A. K. , Gutierrez B. J. , Atkinson J. L. , Ramaiya K. J. , Carter L. J. , Chiu M. T. , Daniels M. A. , Miera M. G. , Maerki S. , Bunch D. P. , Lazarus H. S. , Moore J. , Davis L. C. , Drudge N. , Bobbitt B. J. , Wendel C. L. , Fagan D. F. , Andrews J. , Seyez K. L. , Holmes K. N. , Cadieux L. , Moinfar N. , Friedman S. M. , Williams S. E. , Rehling S. M. , Marsh T. , VandeVelde A. R. , Suiter B. G. , Cooper B. A. , Yeager F. T. , Fox G. M. , Wyrick H. , Hinkel H. A. , Batlle I. R. , Pippin K. , Ainley L. R. , Singh R. S. J. , Perkins S. , Guardado A. , Patel A. K. , Adamo A. M. , Puckett B. S. , Clark D. J. , Flato I. M. , Cohen J. , Kopfer M. , Cleary M. M. , Lee M. S. , Connaughton M. , Tlucek P. S. , Zhang W. , Durrani A. K. , Braverman A. L. , Pulliam B. G. , Gabel D. L. , Reardon D. , Nobel G. S. , Wehmeier J. , Bockius K. R. , Blinder K. J. , Boyd L. K. , Stuart M. A. , Kittleman N. A. , Weeks R. F. , Dang S. , Schremp S. A. , Ibarra A. B. , Pina A. L. , Garza A. , Navarro E. , Villarreal G. L. , Cabrera I. , Salinas N. L. , Patel N. R. , Flores R. R. , Alonso S. , Garza S. , Gonzalez V. H. , Marcus D. M. , Simons E. W. , Ivey K. , Woodward M. , Ortiz S. O. , Bailey T. , Dunn C. J. , Heim E. N. , Stewart M. C. , Varner R. S. , Lamaster S. N. , Rosenthal W. N. , Nakoski B. , Mein C. E. , Belmontes C. A. , Wienecke C. S. , Baskin D. E. , San Roman J. , Castellanos J. , Kirschbaum L. , Adams L. , Chica M. A. , Lane R. G. , Bankston S. , Cloudt S. L. , Martinez V. D. , Lopez V. , Joshi A. S. , Leger A. , Chatham B. R. , Weng C. Y. , Barnett D. B. , Leung E. , Cai J. , Morales J. F. , Baker L. A. , Scholle T. , Volpigno A. , Niffenegger A. , Bentsen B. A. , Scully D. , Richter E. R. , Niffenegger J. H. , Cottrill M. , Lopez M. , Vyas A. P. , Doft B. H. , Stout B. , Foreman C. L. , Diperna D. , Knickelbein J. E. , Walter J. , Olsen K. R. , Stepansky L. , Merlotti L. A. , Forish M. A. , Conrad P. W. , Ostroska P. P. , Bergren R. L. , Menzel A. K. , Klutz A. H. , Stone C. M. L. , Machen D. , Overbey J. C. , Cutshaw K. M. , Raymer L. R. , Rickman L. D. , Hawkins L. H. , Hall M. C. E. , Smith M. , Hamrick M. , Price P. A. , Vincent H. L. , Barrett K. , Fredrick K. J. , Shevchenko L. , Pezda N. F. , Rainey O. P. , Westhouse S. J. , Mohamed S. , Aaberg T. M. , Rosberger D. F. , Wangmo P. , Gyaltshen S. , Villa A. , Workman K. , Oberlander M. , Pereda N. H. , Burgess S. K. , Lara T. M. , Montesclaros C. A. , Vargas C. C. , Mangham C. , Karsaliya G. , Martinez J. A. , Nixon P. A. , le P. V. , Wong R. W. , Young R. C. , Cheek A. G. , Skea B. G. , Waidelich D. C. , Dittman E. A. , Erstad J. N. , Jack L. S. , Wirthlin R. S. , Dessouki A. , Trujillo C. , Chow C. , Dinh D. , Dang H. , Hernandez J. , To K. , He L. , Cummins L. , Montes P. , Fernandez P. D. , Kelley T. , Kuang W. , Malzahn A. K. , DeSilva D. , Vargo H. , Suner I. J. , Traynom J. R. , Henderson K. , Peden M. C. , Munoz S. , Ramsey S. , Wetzel A. , Cook C. R. , Weaver C. , McCluskey J. D. , Wynne K. T. , Kaufman P. L. , Matloff S. , Reynard A. , Connolly B. P. , Hall E. F. , Territo J. , Goole M. , Whelehan M. , Yagoda M. M. , Doran M. J. , Witmer M. T. , Burgess M. , Nelson R. W. , Rose S. J. , Warrington S. , Rodriguez A. , Ortiz C. F. , Figueroa D. , Rodriguez-Roman E. , Mavrofrides E. C. , Kumar J. B. , Haddox M. , Cunningham M. A. , Gomez R. , Houston S. K. S. , Holle T. S. , Neyra A. , Lara D. , Shienbaum G. , Rodriguez J. , Gonzalez M. A. , Garcia P. , Lara W. C. , Castro A. , Santacruz C. , Shaya F. S. , Small K. W. , Jamali A. , Bustamante D. J. , Winje H. A. , Ricks H. , Kwan M. , Miranda M. , Rofagha S. , Aho A. , Haight B. A. , Pallipeedikayil C. C. , Girard C. , Tedstone D. , Johnson E. I. , Velez G. , Larkin J. , Caro J. C. , Rand L. I. , Parry M. A. , Ortega M. D. , Mastrodomenico N. R. , Mykhaylyk O. , Taylor T. , Sweeney T. , Fox A. , Evans B. C. , Wessel C. , Moushon G. W. , Shaw J. , Karrick K. , Bhandari R. , Douglas D. D. , Jois L. M. , Cummings M. K. , Morris R. , Webb R. , Grigorian R. A. , David S. , Ritchey T. , Gerstenblith A. T. , Goldizen A. , Stockman A. L. , Shirey J. , Stambaugh K. , Toomey L. , Glaspell L. , Parnes R. E. , Watkins A. , Ortiz D. , Murillo D. , Dunn G. A. , Qureshi J. A. , Warminski J. D. , Smith P. , Cowart V. E. , Carey A. M. , Oudshoff B. , Glover C. , Handza J. M. , Chiang A. , Kenney B. , Regillo C. , Williams D. , Gonzales E. , Benfield H. , Hsu J. , Grande L. , Huntzberry M. , Millard M. , Merchan A. M. , Hernandez A. , Cardoza C. , Parque K. , MacCumber M. W. , Merrill P. , Montgomery S. E. , Huertas C. , Feinstein E. , Bell G. G. , Kumar G. , Fulgencio J. , Olson J. , Daffron S. , Philibin S. , Tarabishy A. B. , Blair D. , Dunn E. N. , Meyers J. , Goff M. , Spear M. , Summerville A. B. , Hoekzema A. J. , Huynh B. , Thomas B. J. , Carter H. S. , Staman J. A. , Staman J. A. , Yesensky K. D. , Montalvo M. , Charleus M. , Pheng S. B. , Epperson S. , Maximin T. , Hunter A. A. , Beck R. W. , Baptista A. , Beaulieu W. T. , Calhoun C. T. , Constantine S. R. , Correia I. , Dale B. B. , Dupre S. S. , Franklin C. A. , Galusic S. , Huggins M. , Hunter B. L. , Johnson P. A. , Josic K. , Kelly B. , Maguire M. G. , Meadows B. , Melia M. , Preston C. M. , Stockdale C. R. , Zokruah A. , Bhargava S. , Barkmeier A. J. , Baskin D. , Blodi B. , Chew E. , Ferris F. L. , Solomon S. D. , Jaffe G. J. , Bressler N. M. , Lujan B. , Abrams G. , Barnbaum D. R. , Flynn H. , Rudser K. D. , Sternberg P. , Weinstock R. S. , and Wisniewski S. , Cost-Effectiveness of Aflibercept Monotherapy vs Bevacizumab First Followed by Aflibercept if Needed for Diabetic Macular Edema, JAMA Ophthalmology. (2023) 141, no. 3, 268–274, 10.1001/jamaophthalmol.2022.6142, 36729431. 36729431 PMC9896372

[5] Carter P. J. and Lazar G. A. , Next Generation Antibody Drugs: Pursuit of the “High-Hanging Fruit”, Nature Reviews Drug Discovery. (2018) 17, no. 3, 197–223, 10.1038/nrd.2017.227, 29192287. 29192287

[6] Ji L. , Tian H. , Webster K. A. , and Li W. , Neurovascular Regulation in Diabetic Retinopathy and Emerging Therapies, Cellular and Molecular Life Sciences. (2021) 78, no. 16, 5977–5985, 10.1007/s00018-021-03893-9, 34230991. 34230991 PMC8602033

[7] Nentwich M. M. and Ulbig M. W. , Diabetic Retinopathy - Ocular Complications of Diabetes Mellitus, World Journal of Diabetes. (2015) 6, no. 3, 489–499, 10.4239/wjd.v6.i3.489, 25897358. 25897358 PMC4398904

[8] Antonetti D. A. , Silva P. S. , and Stitt A. W. , Current Understanding of the Molecular and Cellular Pathology of Diabetic Retinopathy, Nature Reviews Endocrinology. (2021) 17, no. 4, 195–206, 10.1038/s41574-020-00451-4, 33469209. PMC9053333 33469209

[9] Niu T. , Fang J. , Shi X. , Zhao M. , Xing X. , Wang Y. , Zhu S. , and Liu K. , Pathogenesis Study Based on High-Throughput Single-Cell Sequencing Analysis Reveals Novel Transcriptional Landscape and Heterogeneity of Retinal Cells in Type 2 Diabetic Mice, Diabetes. (2021) 70, no. 5, 1185–1197, 10.2337/db20-0839, 33674409. 33674409

[10] Yazar S. , Alquicira-Hernandez J. , Wing K. , Senabouth A. , Gordon M. G. , Andersen S. , Lu Q. , Rowson A. , Taylor T. R. P. , Clarke L. , Maccora K. , Chen C. , Cook A. L. , Ye C. J. , Fairfax K. A. , Hewitt A. W. , and Powell J. E. , Single-Cell eQTL Mapping Identifies Cell Type-Specific Genetic Control of Autoimmune Disease, Science. (2022) 376, no. 6589, eabf3041, 10.1126/science.abf3041, 35389779. 35389779

[11] Davies N. M. , Holmes M. V. , and Davey S. G. , Reading Mendelian Randomisation Studies: A Guide, Glossary, and Checklist for Clinicians, British Medical Journal. (2018) 362, k601, 10.1136/bmj.k601, 30002074. 30002074 PMC6041728

[12] Li H. , Jiang X. , Xiao Y. , Zhang Y. , Zhang W. , Doherty M. , Nestor J. , Li C. , Ye J. , Sha T. , Lyu H. , Wei J. , Zeng C. , and Lei G. , Combining Single-Cell RNA Sequencing and Population-Based Studies Reveals Hand Osteoarthritis-Associated Chondrocyte Subpopulations and Pathways, Bone Research. (2023) 11, no. 1, 10.1038/s41413-023-00292-7, 37914703. PMC10620170 37914703

[13] Fan C. , Gao Y. , and Sun Y. , Integrated Multiple-Microarray Analysis and Mendelian Randomization to Identify Novel Targets Involved in Diabetic Nephropathy, Frontiers in Endocrinology. (2023) 14, 1191768, 10.3389/fendo.2023.1191768, 37492198. 37492198 PMC10363738

[14] Ren L. , Zhang L. , Bai Y. , Huang C. , Li X. , Ma F. , Mu W. , Yao M. , Jiang C. , Chen X. , Jiang Q. , and Yan B. , Metabolic Stress-Induced Choline Kinase *α* (CHKA) Activation in Endothelial Subpopulation Contributes to Diabetes-Associated Microvascular Dysfunction, Advanced Science. (2025) 12, no. 33, e17045, 10.1002/advs.202417045, 40548950. 40548950 PMC12412581

[15] Wen X. , Han F. , Zhang H. , Luo T. , Jiang J. , Zhang Z. , Zhang T. , Li Y. , Yang L. , Yan W. , He M. , and Long P. , Integrative Single-Cell and Bulk Transcriptomics With Mendelian Randomization Reveals NAD-Related Biomarkers in Diabetic Retinopathy: Insights and Experimental Validation, International Immunopharmacology. (2025) 159, 114949, 10.1016/j.intimp.2025.114949, 40435823. 40435823

[16] Song X. , Ma J. , Zou T. , Lu R. X. , Ji W. , Huang F. , and Yin M. , Cathepsin H Increases the Risk of Diabetic Retinopathy: Evidence From Mendelian Randomization and Bioinformatic Analysis, Diabetology & Metabolic Syndrome. (2025) 17, no. 1, 10.1186/s13098-025-01829-y, 40616157. PMC12231758 40616157

[17] Chen S. , Li B. , Chen L. , and Jiang H. , Uncovering the Mechanism of Resveratrol in the Treatment of Diabetic Kidney Disease Based on Network Pharmacology, Molecular Docking, and Experimental Validation, Journal of Translational Medicine. (2023) 21, no. 1, 10.1186/s12967-023-04233-0, 37308949. PMC10258995 37308949

[18] Spencer B. G. , Estevez J. J. , Liu E. , Craig J. E. , and Finnie J. W. , Pericytes, Inflammation, and Diabetic Retinopathy, Inflammopharmacology. (2020) 28, no. 3, 697–709, 10.1007/s10787-019-00647-9. 31612299

[19] Loh X. Y. , Sun Q. Y. , Ding L. W. , Mayakonda A. , Venkatachalam N. , Yeo M. S. , Silva T. C. , Xiao J. F. , Doan N. B. , Said J. W. , Ran X. B. , Zhou S. Q. , Dakle P. , Shyamsunder P. , Koh A. P. F. , Huang R. Y. J. , Berman B. P. , Tan S. Y. , Yang H. , Lin D. C. , and Koeffler H. P. , RNA-Binding ProteinZFP36L1Suppresses Hypoxia and Cell-Cycle Signaling, Cancer Research. (2020) 80, no. 2, 219–233, 10.1158/0008-5472.CAN-18-2796, 31551365. 31551365

[20] Wang Z. L. , Li B. , Luo Y. X. , Lin Q. , Liu S. R. , Zhang X. Q. , Zhou H. , Yang J. H. , and Qu L. H. , Comprehensive Genomic Characterization of RNA-Binding Proteins Across Human Cancers, Cell Reports. (2018) 22, no. 1, 286–298, 10.1016/j.celrep.2017.12.035. 29298429

[21] Makita S. , Takatori H. , and Nakajima H. , Post-Transcriptional Regulation of Immune Responses and Inflammatory Diseases by RNA-Binding ZFP36 Family Proteins, Frontiers in Immunology. (2021) 12, 10.3389/fimmu.2021.711633, 34276705. PMC8282349 34276705

[22] Kaur P. , Kotru S. , Singh S. , and Munshi A. , miRNA Signatures in Diabetic Retinopathy and Nephropathy: Delineating Underlying Mechanisms, Journal of Physiology and Biochemistry. (2022) 78, no. 1, 19–37, 10.1007/s13105-021-00867-0, 35098434. 35098434

[23] Planel S. , Salomon A. , Jalinot P. , Feige J. J. , and Cherradi N. , A Novel Concept in Antiangiogenic and Antitumoral Therapy: Multitarget Destabilization of Short-Lived mRNAs by the Zinc Finger Protein ZFP36L1, Oncogene. (2010) 29, no. 45, 5989–6003, 10.1038/onc.2010.341, 20802528. 20802528

[24] Youngblood H. , Robinson R. , Sharma A. , and Sharma S. , Proteomic Biomarkers of Retinal Inflammation in Diabetic Retinopathy, International Journal of Molecular Sciences. (2019) 20, no. 19, 10.3390/ijms20194755, 31557880. PMC6801709 31557880

[25] Kinuthia U. M. , Wolf A. , and Langmann T. , Microglia and Inflammatory Responses in Diabetic Retinopathy, Frontiers in Immunology. (2020) 11, 564077, 10.3389/fimmu.2020.564077, 33240260. 33240260 PMC7681237

[26] Cook M. E. , Bradstreet T. R. , Webber A. M. , Kim J. , Santeford A. , Harris K. M. , Murphy M. K. , Tran J. , Abdalla N. M. , Schwarzkopf E. A. , Greco S. C. , Halabi C. M. , Apte R. S. , Blackshear P. J. , and Edelson B. T. , The ZFP36 Family of RNA Binding Proteins Regulates Homeostatic and Autoreactive T Cell Responses, Science Immunology. (2022) 7, no. 76, eabo0981, 10.1126/sciimmunol.abo0981, 36269839. 36269839 PMC9832469

[27] DeMaio A. , Mehrotra S. , Sambamurti K. , and Husain S. , The Role of the Adaptive Immune System and T Cell Dysfunction in Neurodegenerative Diseases, Journal of Neuroinflammation. (2022) 19, no. 1, 10.1186/s12974-022-02605-9, 36209107. PMC9548183 36209107

[28] Adamiec-Mroczek J. and Oficjalska-Młyńczak J. , Assessment of Selected Adhesion Molecule and Proinflammatory Cytokine Levels in the Vitreous Body of Patients With Type 2 diabetes — role of the Inflammatory-Immune Process in the Pathogenesis of Proliferative Diabetic Retinopathy, Graefe′s Archive for Clinical and Experimental Ophthalmology. (2008) 246, no. 12, 1665–1670, 10.1007/s00417-008-0868-6, 18682976. 18682976

[29] Kocabora M. S. , Telli M. E. , Fazil K. , Erdur S. K. , Ozsutcu M. , Cekic O. , and Ozbilen K. T. , Serum and Aqueous Concentrations of Inflammatory Markers in Diabetic Macular Edema, Ocular Immunology and Inflammation. (2016) 24, no. 5, 549–554, 10.3109/09273948.2015.1034804, 26400051. 26400051

[30] Rangasamy S. , McGuire P. G. , and Das A. , Diabetic Retinopathy and Inflammation: Novel Therapeutic Targets, Middle East African Journal of Ophthalmology. (2012) 19, no. 1, 52–59, 10.4103/0974-9233.92116, 22346115. 22346115 PMC3277025

[31] Yue T. , Shi Y. , Luo S. , Weng J. , Wu Y. , and Zheng X. , The Role of Inflammation in Immune System of Diabetic Retinopathy: Molecular Mechanisms, Pathogenetic Role and Therapeutic Implications, Frontiers in Immunology. (2022) 13, 1055087, 10.3389/fimmu.2022.1055087, 36582230. 36582230 PMC9792618

[32] Lawrence T. , Gilroy D. W. , Colville-Nash P. R. , and Willoughby D. A. , Possible New Role for NF-*κ*B in the Resolution of Inflammation, Nature Medicine. (2001) 7, no. 12, 1291–1297, 10.1038/nm1201-1291, 11726968. 11726968

[33] Yan Z. , Wang C. , Meng Z. , Gan L. , Guo R. , Liu J. , Bond Lau W. , Xie D. , Zhao J. , Lopez B. L. , Christopher T. A. , Naik U. P. , Ma X. , and Wang Y. , C1q/TNF-Related Protein 3 Prevents Diabetic Retinopathy via AMPK-Dependent Stabilization of Blood-Retinal Barrier Tight Junctions, Cells. (2022) 11, no. 5, 10.3390/cells11050779, 35269401. PMC8909652 35269401

[34] Hu Y. X. , Zhu R. F. , Qin Y. W. , Zhao X. X. , and Jing Q. , ZFP36L1b Protects Angiogenesis Through Notch1b/Dll4 and Vegfa Regulation in Zebrafish, Atherosclerosis. (2020) 309, 56–64, 10.1016/j.atherosclerosis.2020.07.021, 32882641. 32882641

[35] Sidali A. , Teotia V. , Solaiman N. S. , Bashir N. , Kanagaraj R. , Murphy J. J. , and Surendranath K. , AU-Rich Element RNA Binding Proteins: At the Crossroads of Post-Transcriptional Regulation and Genome Integrity, International Journal of Molecular Sciences. (2022) 23, no. 1, 10.3390/ijms23010096, 35008519. PMC8744917 35008519

[36] Braunger B. M. , Leimbeck S. V. , Schlecht A. , Volz C. , Jägle H. , and Tamm E. R. , Deletion of Ocular Transforming Growth Factor *β* Signaling Mimics Essential Characteristics of Diabetic Retinopathy, American Journal of Pathology. (2015) 185, no. 6, 1749–1768, 10.1016/j.ajpath.2015.02.007, 25857227. 25857227

[37] Dagher Z. , Gerhardinger C. , Vaz J. , Goodridge M. , Tecilazich F. , and Lorenzi M. , The Increased Transforming Growth Factor-*β* Signaling Induced by Diabetes Protects Retinal Vessels, American Journal of Pathology. (2017) 187, no. 3, 627–638, 10.1016/j.ajpath.2016.11.007, 28162229. 28162229 PMC5397667

[38] Wang M. , Sheng K. J. , Fang J. C. , Zhao H. , Lu S. M. , Liu Z. Y. , and Chen B. T. , Redox Signaling in Diabetic Retinopathy and Opportunity for Therapeutic Intervention Through Natural Products, European Journal of Medicinal Chemistry. (2022) 244, 114829, 10.1016/j.ejmech.2022.114829, 36209631. 36209631

[39] Yuan S. , Zhai Y. , Tao T. , Zhang X. , Bashir G. , Li G. , Wang G. , and Wu S. , Conflicting Roles of ZFP36L1 in Regulating the Progression of Muscle Invasive Bladder Cancer, Frontiers in Molecular Biosciences. (2022) 9, 10.3389/fmolb.2022.687786, 35359594. PMC8962643 35359594

[40] Sinha S. , Dutta S. , Datta K. , Ghosh A. K. , and Mukhopadhyay D. , Von Hippel-Lindau Gene Product Modulates TIS11B Expression in Renal Cell Carcinoma, Journal of Biological Chemistry. (2009) 284, no. 47, 32610–32618, 10.1074/jbc.M109.058065, 19801654. 19801654 PMC2781675

[41] Snyder B. L. and Blackshear P. J. , Clinical Implications of Tristetraprolin (TTP) Modulation in the Treatment of Inflammatory Diseases, Pharmacology & Therapeutics. (2022) 239, 108198, 10.1016/j.pharmthera.2022.108198, 35525391. 35525391 PMC9636069

[42] Tarling E. J. , Clifford B. L. , Cheng J. , Morand P. , Cheng A. , Lester E. , Sallam T. , Turner M. , and de Aguiar Vallim T. Q. , RNA-Binding Protein ZFP36L1 Maintains Posttranscriptional Regulation of Bile Acid Metabolism, Journal of Clinical Investigation. (2017) 127, no. 10, 3741–3754, 10.1172/JCI94029, 28891815. 28891815 PMC5617661

[43] Shi J. X. , Su X. , Xu J. , Zhang W. Y. , and Shi Y. , MK2 Posttranscriptionally Regulates TNF-*α*-Induced Expression of ICAM-1 and IL-8 via Tristetraprolin in Human Pulmonary Microvascular Endothelial Cells, American Journal of Physiology-Lung Cellular and Molecular Physiology. (2012) 302, no. 8, L793–L799, 10.1152/ajplung.00339.2011, 22268119. 22268119

[44] Brooks S. and Rigby W. , Post-Transcriptional Regulation of Tumor Necrosis Factor Alpha Expression, Arthritis Research & Therapy. (2004) 6, no. 3, 10.1186/ar1349.

[45] Pham M. H. T. , Bonello G. B. , Castiblanco J. , le T. , Sigala J. , He W. , and Mummidi S. , The rs1024611 Regulatory Region Polymorphism Is Associated With CCL2 Allelic Expression Imbalance, PLOS One. (2012) 7, no. 11, e49498, 10.1371/journal.pone.0049498, 23166687. 23166687 PMC3500309

[46] Cao H. , Urban J. F. , and Anderson R. A. , Insulin Increases Tristetraprolin and Decreases VEGF Gene Expression in Mouse 3T3-L1 Adipocytes, Obesity. (2008) 16, no. 6, 1208–1218, 10.1038/oby.2008.65, 18388887. 18388887

[47] Han X. , Gharahkhani P. , Hamel A. R. , Ong J. S. , Rentería M. E. , Mehta P. , Dong X. , Pasutto F. , Hammond C. , Young T. L. , Hysi P. , Lotery A. J. , Jorgenson E. , Choquet H. , Hauser M. , Cooke Bailey J. N. , Nakazawa T. , Akiyama M. , Shiga Y. , Fuller Z. L. , Wang X. , Hewitt A. W. , Craig J. E. , Pasquale L. R. , Mackey D. A. , Wiggs J. L. , Khawaja A. P. , Segrè A. V. , 23andMe Research Team , International Glaucoma Genetics Consortium , and MacGregor S. , Large-Scale Multitrait Genome-Wide Association Analyses Identify Hundreds of Glaucoma Risk Loci, Nature Genetics. (2023) 55, no. 7, 1116–1125, 10.1038/s41588-023-01428-5, 37386247. 37386247 PMC10335935

