# Supplementary material for: Multiomics Integration Prioritizes ZFP36L1 as a Candidate Susceptibility Gene Associated With Inflammatory and Angiogenic Pathways in Diabetic Retinopathy
Source: J Diabetes Res. 2026 Jun 19;2026:4312504. doi: 10.1155/jdr/4312504 (PMC13280981; doi:10.1155/jdr/4312504)
Supplement: Supplementary file 1 — Supporting Information 1 Table S1: Instrument characteristics and sensitivity metrics for Mendelian randomization analyses of the eight genes associated with diabetic retinopathy. [file JDR-2026-4312504-s001.docx]

Supplementary Table 1 Instrument characteristics and sensitivity metrics for Mendelian randomization analyses of the eight genes associated with diabetic retinopathy.

| IVs | SNP | | IVW method (multiplicative random-effects) | | MR-Egger regression analysis | | Cochran’s Q test | |
| --- | --- | --- | --- | --- | --- | --- | --- | --- |
|  | Number of SNPs | SNPs | OR | P-value | Intercept | P-value | Q statistic | P-value |
| *CA2* | 8 | rs10852622, rs10876550, rs114694170, rs11702779, rs1496531, rs2930553, rs505401, rs7973618 | 0.944 | 0.010 | 0.000 | 0.985 | 1.863 | 0.967 |
| *CTSH* | 3 | rs62013198, rs6495338, rs67674051 | 1.069 | 0.001 | 0.011 | 0.704 | 0.851 | 0.654 |
| *GLUL* | 4 | rs10752870, rs115388715, rs149007767, rs56330463 | 1.127 | 0.027 | 0.010 | 0.751 | 5.179 | 0.159 |
| *IER3* | 8 | rs1128175, rs114708313, rs1264372, rs149110519, rs2233966, rs56330463, rs7210990, rs77526211 | 1.211 | 0.000 | 0.013 | 0.537 | 27.222 | 0.000 |
| *LPCAT1* | 3 | rs10052016, rs149110519, rs36976 | 0.871 | 0.030 | -0.012 | 0.921 | 2.693 | 0.260 |
| *MXI1* | 3 | rs10509912, rs2855469, rs592423 | 0.923 | 0.019 | -0.020 | 0.408 | 1.933 | 0.38 |
| *PDGFRB* | 3 | rs2524054, rs55908509, rs7944004 | 0.818 | 0.004 | -0.003 | 0.947 | 0.477 | 0.788 |
| *ZFP36L1* | 3 | rs1884808, rs7210990, rs8078723 | 1.156 | 0.002 | 0.012 | 0.562 | 0.753 | 0.686 |

IVs, instrumental variables; IVW, inverse‐variance weighted.
